# Catch Me If You Can: The Link between Autophagy and Viruses

**DOI:** 10.1371/journal.ppat.1004685

**Published:** 2015-03-26

**Authors:** Nicholas J. Lennemann, Carolyn B. Coyne

**Affiliations:** Department of Microbiology and Molecular Genetics, University of Pittsburgh School of Medicine, Pittsburgh, Pennsylvania, United States of America; University of Florida, UNITED STATES

## What Is Autophagy?

**Fig 1:**
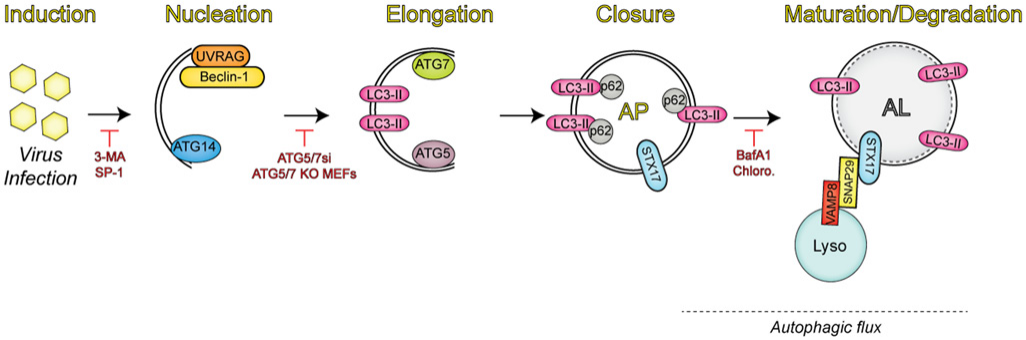
Overview of the autophagic pathway. Upon infection, viruses trigger the induction of autophagy through a number of mechanisms. Autophagy regulators (i.e., Beclin-1, UVRAG, and ATG14) function in membrane nucleation to form the double-membraned phagophore, which can be blocked via addition of pharmacological inhibitors (3-MA, spautin-1 [SP-1]). Additional autophagy-related proteins (ATG7 and ATG5) mediate the elongation step, in which the phagophore begins to expand until it closes around the material targeted for degradation by sequestration proteins, such as SQSTM/p62. Inhibition of this event is commonly performed through the expression of small interfering RNAs (siRNAs) targeting the autophagic components involved in this process. The completed autophagosome (AP) is then able to fuse with lysosomes (Lyso) via the soluble N-ethylmaleimide-sensitive factor attachment protein receptor (SNARE) complex consisting of syntaxin 17 (STX17), SNAP29, and VAMP8. The engulfed contents are then degraded, along with the inner membrane in the newly formed autolysosome (AL), in a process termed autophagic flux. Vesicle acidification inhibitors have been used to block degradation in the AL, given that lysosomal proteases are only active at low pH.

Autophagy is a process that mediates the degradation of cytoplasmic material, such as damaged organelles and protein aggregates, to maintain cellular homeostasis (Fig. 1) [1]. The autophagic pathway begins with the sequestration of organelles and portions of the cytoplasm via a double-membrane termed the isolation membrane (or phagophore), which can be derived from several cellular compartments (including the endoplasmic reticulum [ER], Golgi complex, ER-Golgi intermediate compartment [ERGIC], mitochondria, or ER-mitochondria associated membranes [MAMs], as well as the plasma membrane) [2]. The isolation membrane expands to completely envelop the isolated contents in a double-membrane vesicle called the autophagosome, which then undergoes maturation through fusion with lysosomes to form autolyosomes [3]. A hallmark of canonical autophagy (or “macroautophagy”) is autophagic flux, in which lysosomal enzymes degrade the contents within the autolysome. Alternatively, early/late endosomes can fuse with autophagosomes, forming amphisomes that can then mature to autolysosomes, in which both endosomal and autophagosomal contents are degraded.

## Autophagy during Viral Infections: A Blessing in Disguise?

Autophagy is thought to be an ancient process that may have evolved to combat infection by a number of intracellular pathogens [4]. Work from our laboratory has shown that autophagy is induced by placental-derived microRNAs (miRNAs) carried in exosomes to attenuate viral infections in non-placental cells [5]. Furthermore, human placental trophoblasts, the specialized cells that comprise the placenta, exhibit high levels of autophagy themselves, which might contribute to their resistance to viral infections [5]. Autophagy is often a constitutive process that occurs at basal levels to maintain cellular homeostasis. However, autophagy can also be induced in response to cellular stresses such as nutrient deprivation, the unfolded protein response (UPR), or oxidative stress [6]. Given the amount of stress a viral infection elicits within a host cell, it is not surprising that this event often triggers autophagy, which can function as either a proviral or antiviral pathway, depending on the viral inducer.

Autophagosome formation requires extensive membrane remodeling, which is also induced during the replication of positive-strand RNA viruses. Indeed, many positive strand RNA viruses including picornaviruses and flaviviruses induce the autophagic process during their replicative life cycles to generate the membranes necessary for the biogenesis of their replication organelles. In addition, a diverse array of other viruses also induce autophagy (reviewed in [4]), including members of the paramyxoviridae, orthomyxoviridae, togaviridae, and herpesviridae. During infection, viruses (and/or virally-encoded proteins) can be targeted for degradation by induction of the autophagic pathway as a means to control their replication. For example, Sindbis virus capsid protein is targeted to autophagosomes and degraded during the process of autophagic flux, which functions to suppress new virion formation [7]. Interestingly, several herpesviruses express proteins that directly inhibit the formation of autophagosomes, indicating that these viruses may have evolved strategies to evade the degradative nature of the autophagic pathway [4]. Indeed, decreased neurovirulence is observed in mice infected with a mutant herpes simplex virus-1 that is unable to block autophagosome formation [8].

Host cells induce the formation of autophagosomes through a variety of mechanisms and in response to several events during the viral life cycle. During antiviral signaling, engagement of vesicular stomatitis virus by the pattern recognition receptor Toll-7 at the cell surface induces an autophagy-dependent innate immune response mediated by phosphatidylinositol 3-kinase (PI3K)-Akt-signaling in *Drosophila* that limits viral replication [9,10]. In addition, autophagy controls Rift Valley fever virus (RVFV) replication in both flies and mammals via toll-like receptor signaling [11]. Some viruses induce autophagy at the earliest stages of their life cycles—measles virus (MeV) induces autophagy through binding to CD46, a cell surface receptor required for MeV entry [12]. At later stages of infection, expression of the MeV C protein is sufficient to induce a second wave of autophagy via interaction with immunity-associated GTPase family M (IRGM), a known regulator of autophagy [13]. Consistent with this, several other viruses have been shown to induce the formation of autophagosomes at late stages of their replicative life cycles, often as a consequence of the dramatic increases in protein production resulting from viral gene expression. For example, it has been reported that hepatitis C virus (HCV) induces autophagy through interaction with IRGM and ER stress by triggering the UPR [13,14]. The autophagic machinery has been shown to be involved in the initial translation of HCV RNA, but not maintenance of viral replication [15]. Several other viruses benefit from the induction of autophagy and have evolved strategies to directly manipulate the autophagic machinery in order to enhance their replication and/or egress.

## How Do Viruses Benefit from Autophagy?

Enteroviruses have been extensively studied for the beneficial role that autophagy plays in their replicative life cycles. Replication of several members of the enterovirus family including poliovirus (PV) and coxsackievirus B (CVB) is enhanced by virus-induced autophagy [16,17]. Both PV and CVB infection results in the formation of autophagosome-like double-membraned vesicles, which serve as scaffolding for viral RNA replication [16,18]. Thus, these viruses usurp the autophagic pathway to provide the membranes necessary for these replication sites. It has been suggested that acidification of vesicles, potentially maturing autophagosomes, during PV infection promotes maturation of virions to the infectious particles [19]. However, autophagy-dependent degradation is not required for PV replication [19]; thus, maturation of virions does not depend on the degradative capacity of autolysosomes. Furthermore, a recent report suggests that PV takes advantage of autophagy-dependent exocytosis to aid in the release of virions into the extracellular space prior to viral-mediated lysis of the host cell in a process termed autophagosome-mediated exit without lysis (AWOL) [20]. Consistent with this finding, CVB can also be released in microvesicles containing the autophagosomal marker, microtubule-associated protein 1A/1B-light chain 3 (LC3-II) [21]. Unlike PV, CVB infection has been suggested to block the formation of autolysosomes, which has been hypothesized to be a mechanism to evade lysosomal degradation [17]. However, the role autophagic flux might play in CVB replication remains unclear. Although the precise mechanism(s) by which PV and CVB induce the autophagic pathway and manipulate select aspects of autophagy require further delineation, it is clear that autophagy plays an important role in the enhancement of enterovirus infection, most likely independent of autophagic flux.

In direct contrast to enteroviruses, MeV replication benefits from autophagic flux. Despite the completion of the autophagic maturation process, MeV proteins are not targeted for degradation by autophagosome-lysosome fusion [22]. Rather, induction of autophagy during MeV infection serves to prevent the induction of cell death. Given the role of autophagy in maintaining cellular homeostasis, it may not be surprising that infection activates this pro-survival pathway to prolong the life of the cell in order to generate a maximal number of progeny virions.

Similar to PV, autophagy also benefits the maturation of dengue virus (DENV) particles [23]. After replication in ER-associated vesicles, DENV particles are assembled in the ER and transported through the secretory pathway prior to their release into the extracelluar space. Within the Golgi complex, one of the viral surface glycoproteins, prM, is cleaved by the resident trans-Golgi protease furin, which results in the generation of infectious viral particles [24]. Inhibition of autophagy decreases the specific infectivity of extracellular virions, which corresponds with the observed decrease in prM cleavage on virions released from the cell.

Unlike enteroviruses, DENV directly benefits from a specific autophagy-dependent process termed “lipophagy” to increase its replication. Lipophagy represents an alternative pathway of lipid metabolism and is mediated by the degradative nature of autophagic flux. During DENV infection, lipid droplets colocalize with autolysosomes, which correlates with a decrease in cellular triglycerides [25]. Consequently, the released free fatty acids from lipid droplets are processed in the mitochondria via β-oxidation, resulting in an increase in cellular ATP. It has been proposed that this increase in ATP provides the source of energy required to facilitate the various processes involved in DENV replication. Thus, DENV infection induces autophagy-dependent lipid metabolism (“lipophagy”) to facilitate its replication. In another study, it was proposed that inhibition of autophagosome formation dramatically limits, but does not completely abolish, DENV replication [26]. Thus, either DENV does not absolutely require autophagy for replication or there are additional, yet to be defined pathways that regulate autophagosome formation during infection.

## What Mechanisms Mediate Viral Manipulation of Autophagy?

A recent report has shown that human parainfluenza virus type 3 (HPIV3) infection induces the accumulation of cytoplasmic autophagosomes by the direct inhibition of autophagic flux [27]. Autolysosome formation is dependent on the interaction of three SNAREs. Fusion of autophagosomes with lysosomes is dependent on binding of the adaptor protein SNAP29 with syntaxin-17, located on the autophagosomal membrane, and the late endosome/lysosome membrane protein VAMP8 [28]. Expression of the HPIV3 phosphoprotein (P) inhibits the interaction of syntaxin-17 with SNAP29 by specifically interacting with both SNARE domains of SNAP29, thus directly inhibiting the ability of autophagosomes to fuse with lysosomes [27]. The inhibition of autolysosome formation results in an increase in extracellular virion production by a currently unknown mechanism [27].

Accumulation of autophagosomes is also observed during influenza A virus (IAV) infection [29]. The viral matrix 2 (M2) ion-channel protein contains a highly conserved LC3-interacting region (LIR) that mediates its interaction with the autophagosome-associated component LC3. Upon infection, LC3 relocalizes to the plasma membrane in an M2 LIR-dependent manner. Disruption of M2-LC3 interactions decreases filamentous virion budding and stability. Thus, in addition to modulating cell death pathways [30], IAV infection may subvert autophagy to enhance transmission to new cells and/or hosts by increasing virion stability.

Human immunodeficiency virus-1 (HIV-1) infection also results in the subversion of the autophagic pathway to facilitate new virion formation [31]. The HIV-1 precursor protein Gag interacts with LC3, which facilitates the processing of Gag into the virion core structural proteins. Furthermore, the HIV-1 accessory protein Nef blocks autophagosome maturation through its interaction with Beclin-1, a central regulator of autophagy. Deletion of Nef from the viral genome leads to autophagy-dependent degradation of the viral capsid protein p24. Therefore, HIV-1 infection requires the induction of autophagy for Gag processing, but inhibits the degradative capacity of this pathway to increase virion production.

## Perspectives

Despite our appreciation that many viruses utilize autophagy in a proviral manner, relatively little is known regarding the specific mechanisms by which viruses induce autophagy and/or manipulate this pathway for their gain. In addition, the term “autophagy” most commonly refers to macroautophagy, a non-selective process that mediates bulk degradation, but there are many selective forms of autophagy that are named after the degradation targets, including mitophagy (mitochondria); pexophagy (peroxisomes); aggrephagy (protein aggregates); glycophagy (glycogens); lipophagy (lipids); and, recently, ER-phagy (endoplasmic reticulum) (reviewed in [32]). It will, therefore, be important to determine if these specialized autophagic processes are exploited by viruses to enhance their infection, as has been described for DENV and lipophagy. Recent work from our laboratory has identified a novel regulator of a noncanonical autophagic pathway that functions independent of the core initiation machinery to limit enterovirus infection [33], suggesting that enteroviruses might target noncanonical forms of autophagy to maximize their replication. In addition, the recent identification of specific regulators of autophagosome maturation, such as syntaxin-17, SNAP29, and VAMP8, presents an exciting opportunity to further define the role of this critical autophagic process in limiting and/or enhancing viral infections. The identification of specific regulators of the various stages of autophagy may also serve to clarify seeming inconsistencies in the literature, which were often published prior to the identification of these regulators. Although the field of autophagy is rapidly progressing, there is still much to be learned regarding the specific molecules that regulate this tightly controlled pathway and the mechanisms by which viruses target these molecules to facilitate their replication.
